# An Evaluation of the New Zealand *SilverTech* Smartphone Course for Older Adults

**DOI:** 10.1177/07334648241306941

**Published:** 2024-12-09

**Authors:** Chrystal Jaye, Rebecca McLean, Janet Lin, Kristen Beardsmore, Debbie George

**Affiliations:** 1 Department of General Practice and Rural Health, 2495 University of Otago, Dunedin, New Zealand; 2 Department of Population Health, 2495 University of Otago, Christchurch, New Zealand; 3 Otago Medical School, 2495 University of Otago; 4 Age Concern Otago, Dunedin, New Zealand

**Keywords:** digital literacy, education, evaluation, learning, New Zealand, smartphones

## Abstract

In contemporary society, where digital literacy is an essential skill, older adults can face disadvantages because they frequently have lower levels of digital literacy than younger cohorts. This research evaluated the effectiveness of a smartphone course developed by Age Concern Otago (a New Zealand non-government organization), with the aim of improving digital literacy among older adults. This was achieved using a pre- and post-course survey study design. A total of 98 participants, aged above 60, from thirteen *SilverTech* courses (four two-hourly sessions) completed pre-surveys in the first session and post-surveys in the last session of each course. The results showed that participants’ smartphone digital literacy was improved across all evaluated indices, and feedback for the courses was positive. These findings illustrate that the *SilverTech* courses are an effective means of improving the digital literacy of older adults and thus fostering digital inclusion in New Zealand.

**What this paper adds**

• Older adults are motivated to use smartphones because they understand their potential and utility in improving their daily lives.
• If smartphones have disability-friendly functions and design features, older adults will use these if taught how to.
• The Age Concern Otago *SilverTech* course is a well-designed, accessible, and effective community run program improving smartphone digital literacy for older adults.

**Applications of study findings**

• Age Concern Otago could confidently extend the *SilverTech* program across New Zealand.
• Governments need to support free or low-cost smartphone courses for older adults run by community organizations such as Age Concern Otago in order to improve digital inclusion.
• Smartphone developers should consider the needs of the growing market demographic of older adults in the design of smartphones.

## Introduction

The World Health Organization defines active aging in terms of being able to optimize opportunities for health, participation, and safety so that quality of life improves as an individual ages (Tirado-Morueta et al., 2018; World Health Organization, 2002). Yet older adults experience several individual- and societal-level disadvantages because they frequently have lower levels of digital literacy than younger cohorts (Blažič & Blažič, 2020). As banking, public, and health services have been digitized, digital literacy has become an essential skill in contemporary society (Barrie et al., 2021; Citizens Advice Bureaux New Zealand, 2020; Martínez-Alcalá et al., 2018; Rose et al., 2020). The increasing use of digital technologies in healthcare services such as telehealth and health provider digital portals illustrate the impacts of digital exclusion (Choi & DiNitto, 2013; Oh et al., 2021; Tsai et al., 2017; Zolbin et al., 2022). Older adults with low digital literacy are less likely to use electronic resources such as health and medical services (Blažič & Blažič, 2020; Susło et al., 2018; Zolbin et al., 2022)—issues that were highlighted during the COVID-19 pandemic. The digitization of critical services, including banking and healthcare, has the potential to exacerbate existing health inequities (Bejarano, 2022) and increase societal marginalization for those who lack digital resources and/or competencies (Tsai et al., 2017).

### Digital Exclusion, Inclusion, and Literacy

Factors contributing to digital exclusion include infrastructural factors (such as network cover); access issues (such as affordability); and intrapersonal components that include motivation to use technology, available supports, and functional limitations (such as cognitive decline, spatial orientation, and arthritis) (Lin cited in Castilla et al., 2018; Holgersson et al., 2021; McDonough & Kingsley, 2015; Rose et al., 2020; Tirado-Morueta et al., 2018; Tsai et al., 2017). One issue that particularly affects older adults and those with disabilities lies in device and software designs that are oriented around the needs and abilities of younger cohorts (such as digital dexterity, technological fluency, and visual acuity) (Delello & McWhorter, 2017).

Digital inclusion for members of society is considered to be achievable when four criteria are met: being motivated to use the internet; having access to the internet; possessing core digital skills; and trusting online services (Digital Inclusion Research Group, 2017). Digital literacy has been defined in terms of possessing the necessary skills required to participate fully in the contemporary digital society (Barrie et al., 2021; Martínez-Alcalá et al., 2018). These include mechanical and navigational, technical, and cognitive/conceptual competencies (Schreurs et al., 2017). One conceptual barrier for older adults entering cyber space is the bewildering array and presentation of information, particularly for a generation more familiar with analogue media such as printed material or television (Castilla et al., 2018). The hypertext link format of the Internet is one structure that can be confusing and disorienting to older users (Castilla et al., 2018). Another conceptual barrier to technological adoption is anxiety about cybersecurity and fear of being scammed (Holgersson et al., 2021).

Although internet access by older adults is increasing (and NZ has one of the highest usage rates in the world [Kemp, 2023; Koopman-Boyden & Reid, 2009]), this group continues to demonstrate lower levels of digital literacy than younger cohorts (Barrie et al., 2021; Flauzino et al., 2020; Lips et al., 2020; Tsai et al., 2017), an age-based digital gulf (Tirado-Morueta et al., 2018) described by Schreurs et al. (2017) as a “gray divide.” In NZ many rural regions do not yet have high speed fiber networks (Mitchell, 2024), and older rural-dwelling adults may experience additional problems (Castilla et al., 2018). It has been suggested that digital exclusion reflects the demographic inequities that exist off-line (Tirado-Morueta et al., 2018). For example, in the NZ context, Māori and Pacific Island people are disproportionately impacted by digital exclusion and this is likely to be exacerbated among older Māori and Pacific Island cohorts (Citizens Advice Bureaux New Zealand, 2020; Digital Inclusion Research Group, 2017).

Older adults are not a homogenous group (Barrie et al., 2021). Socio-demographics, past employment, education, and gender are known to impact digital literacy levels and digital skills (Choi & DiNitto, 2013; Koopman-Boyden & Reid, 2009; Tirado-Morueta et al., 2018). Levels of digital literacy have been shown to be inversely linked with levels of anxiety in using technology, while experience with digital technology leads to increased confidence and decreased anxiety about using digital technologies (Castilla et al., 2018; Susło et al., 2018).

### Benefits of Digital Literacy

Numerous benefits from improving digital literacy for older adults have been described. These encompass health impacts (Barrie et al., 2021; Koopman-Boyden & Reid, 2009) including motor and cognitive skills (Delello & McWhorter, 2017; Flauzino et al., 2020; Tsai et al., 2017), self-management and health monitoring (Marcelino et al., 2015; Zolbin et al., 2022), quality of life (Schreurs et al., 2017; Tsai et al., 2017; Zhao et al., 2020), social participation (Barrie et al., 2021; Castilla et al., 2018; Flauzino et al., 2020; Koopman-Boyden & Reid, 2009; Martínez-Alcalá et al., 2018; Oh et al., 2021; Tirado-Morueta et al., 2018), independence (Barrie et al., 2021), empowerment (Zolbin et al., 2022), subjective well-being (Flauzino et al., 2020; Koopman-Boyden & Reid, 2009; Tirado-Morueta et al., 2018), learning and information sharing (Oh et al., 2021; Tirado-Morueta et al., 2018), online shopping (Lips et al., 2020), economic participation and benefits (Digital Inclusion Research Group, 2017; Sung, 2016), and entertainment.

### Digital Literacy Education

Previous research illustrates that older adults are capable of learning and improving their digital competencies (Delello & McWhorter, 2017; Marcelino et al., 2015; Martínez-Alcalá et al., 2018). Providing suitable digital literacy education to older adults requires consideration of their unique andragogical needs (Merriam & Kee, 2014). Tan (2018, cited in Flauzino et al., 2020) recommends that teaching and learning models be strongly learner centered, while Martίnez-Alcalá et al. (2018) observed that education environments for older adults should support previous experience and diverse learning styles. Training programs should avoid top-down ageist attitudes and condescension towards older adults (Barrie et al., 2021; Martínez-Alcalá et al., 2018). Teachers also need to account for older adults’ sensorial deficits and support their autonomy during the learning process (Flauzino et al., 2020; Martínez-Alcalá et al., 2018; Oh et al., 2021). In one study, training courses that incorporated a combination of written and verbal instruction, simplified materials, explanation of technological symbols, continuous repetition, and practice experience were considered most effective from both learners’ and teachers’ perspectives (Flauzino et al., 2020). The willingness to explore, have fun, and “play around” on digital devices is essential to the process of improving digital literacy (Blažič & Blažič, 2020; Tsai et al., 2017). New technologies are more likely to be adopted by older adults when the usefulness is apparent and relevant and when they have family support (Martínez-Alcalá et al., 2018).

A New Zealand report indicates that only around 20% of digital inclusion initiatives have been evaluated and that there is no agreement on what digital inclusion outcomes should be measured and how this should occur (Department of Internal Affairs, 2022). One systematic review on the measurement of digital literacy among older adults noted that digital literacy involves several interrelated skillsets and that many instruments in their review tended to measure attitudes towards technology and not the digital aptitude itself (Oh et al., 2021). Findings from another systematic review reported a noticeable difference in post-intervention data (Zolbin et al., 2022). With regard to smartphones, Lin and Ho (2020) argued that the impact of age-related motor declines on older adults’ ability to use smartphone technology is poorly understood, while Roque and Boot (2018) observed that any evaluation requires the assessment of initial proficiencies to provide a baseline. Research conducted by the Pew Research Centre (Anderson & Perrin, 2017; Faverio, 2022) indicates that the differences in adoption of smartphones between youngest and oldest adult users are decreasing. However, the design interface of smartphones and tablets (particularly touchscreens) can be difficult for older adults with arthritis, vision and/or psychomotor impairments, and device size can cause discomfort (Choi & DiNitto, 2013; Lin & Ho, 2020; Lu et al., 2017; McDonough & Kingsley, 2015; Susło et al., 2018; Tsai et al., 2017). These factors explain the lower uptake of smartphone technology among older adults (Blažič & Blažič, 2020; Lin & Ho, 2020; Lu et al., 2017; Rosales & Fernández-Ardèvol, 2019; Zhao et al., 2020). Despite these limitations, smartphones and tablets have the advantage over personal computers of being relatively cheap and already internet enabled (Roque & Boot, 2018; Zhao et al., 2020). Smartphone health apps can support self-management (including appointment and medication reminders, and chronic condition monitoring), and features such as GPS tracking offer safety benefits (Lin & Ho, 2020).

The rapid uptake and increasing capabilities of smartphones and tablet devices pose ongoing challenges for older adults. The continuous development of new technologies requires users to keep up and this disadvantages older adults, and there is cynicism as to whether the digital divide can ever be closed (Sung, 2016). It is clear that further research is required in order that levels of digital literacy in older adults become better understood (Lin & Ho, 2020; Oh et al., 2021; Zolbin et al., 2022).

### Study Context

New Zealand research conducted in 2017 indicated that 10% of the 65–74 age group did not use the internet compared to three percent of the under 65 age group. Twenty-five percent of 75–84 year olds did not use the internet, and this figure increased to 50% for those aged 85 and over (Lips et al., 2020). Not only are older New Zealand adults less likely to use the internet but their use of the internet is also narrower—the most popular activities for those aged 65 and over are emailing and accessing information about goods and services (Davidson cited in Lips et al., 2020).

The present study was conducted by researchers affiliated to the CARE Research Theme, University of Otago, in collaboration with Age Concern Otago (ACO), a national non-government organization (NGO) committed to the welfare of older adults in New Zealand. In this article, we describe the impact of ACO’s *SilverTech* smartphone training program for older adults. The research collaboration arose from ACO’s desire to evaluate the effectiveness of the course with a view to extending it across Otago (including rural areas) and ultimately expanding *SilverTech* nationally across New Zealand. The research question reflected this: what is the impact of the *SilverTech* course on older adults’ ability to use smartphone technology? The research objectives included assessing the competencies and skills older adults gained from the course, and its impact on their confidence to engage with the digital world.

## Methods

### Study Design and Ethical Approval

This observational study employed a within-subject method with a pre- and post-survey study design. A within-subject research design is used when data measurements are taken from the same group of participants at different time intervals—such as before and after an educational intervention (Simkus, 2023). The study received ethical approval from the University of Otago Human Ethics Committee (D21-339).

### The SilverTech Course Design

The *SilverTech* course was designed and piloted by ACO in response to an unpublished survey the organization conducted after the 2020 COVID-19 Lockdowns which revealed a strong desire by respondents to improve their digital literacy. The courses were free-of-charge, and advertised both online, through posters in local community, and in a weekly newspaper delivered free to all households in the greater Dunedin region. The aims of the *SilverTech* course were to provide an introductory digital literacy training course for older adults (65+ years) that would improve technical competencies and confidence in using a smartphone. The course was aligned to the recommendations of Martίnez-Alcalá et al. (2018) that the content of the course be personally and socially useful to older adult participants; with a focus on teamwork, interaction, and support; and the aim to promote autonomy and social inclusion. All courses took place in venues that were easily accessible to people with mobility issues. Each *SilverTech* course consisted of four weekly two-hour sessions and was limited to eight participants. The course supported participants learning on Android or Apple devices and covered the following aspects over four sessions: setting up and navigating devices, setting up contact lists, texting, emailing, managing apps (including banking and health apps), managing Wi-Fi/data, using social media and entertainment (music and videos), and online safety. Each class had one trained facilitator, and the small class size ensured that attendees received individual attention from that facilitator. Trainers demonstrated how to use an Android and Apple smartphone for each of the learning outcomes. Table 1 lists the learning outcomes for each session. Participants then had the opportunity to practice the skill on their own device and receive feedback and assistance during class time. The demonstration and practice were supported by take home learning materials (Android or Apple based) detailing each skill.

**Table 1.** Learning Outcomes of Each Session of SilverTech Course.

Weekly session	Learning outcomes
1	• Identify an app icon
	• Identify the different physical buttons
	• Identify the different screen navigation buttons
	• Know what finger gestures are available
	• Know how to change various settings
	• Know your way around the keyboard
	• Know the difference between Wi-Fi and mobile data
2	• Set up a password
	• Add emergency contacts
	• Send a text
	• Send a group text
	• Take a photo/ selfie
	• Send a text with a photo
	• Make a call
	• Make a video call
3	• Set a world clock
	• Set an alarm
	• Create a calendar event
	• Create a note
	• Send a photo by email
	• Search maps using voice
4	• Download an app
	• Move an app around your screens
	• Delete /uninstall an app
	• Put apps in a folder
	• Spot a scam

### Recruitment and Data Collection

Pre- and post-course survey data were collected from participants attending a total of 13 *SilverTech* courses run at six locations in Otago, New Zealand, from February to June 2022. In the first session of each *SilverTech* course, attendees were informed about the research study and given the opportunity to participate. Written informed consent was collected from those who agreed to participate prior to completion of paper pre-course surveys. At the final session of the course, participants were asked to complete paper post-course surveys. These were completed individually and handed in at the course venue. A total of 122 participants completed pre-course surveys. Ninety-eight of these participants completed post-course surveys (follow-up rate = 74%) while 24 dropped out for undocumented reasons.

#### Surveys

Pre- and post-course surveys were used to measure participants’ digital literacy for smartphone use *before* and *after* attending the *SilverTech* course, respectively. These drew upon previously published questionnaires (Roque & Boot, 2018). The pre-course survey contained 37 items, divided into two domains: general demographics and digital literacy questions. The post-course survey contained 33 items, divided into two domains: digital literacy questions (the same as those in pre-course survey), and additionally, evaluation of course content.

#### Measures

Variables under each domain are described below.

##### General Demographic

The pre-course survey contained a demographic domain: gender (male or female), age (61–65, 66–70, 71–75, 76–80, 81–85, or 86+), ethnicity (New Zealand European, Māori, Chinese, European, Pacific Island, or others), and living situation (living alone or living with others).

##### Digital Literacy Assessment

General assessment: Four questions on the first page of both pre- and post-course surveys asked participants to self-assess their general smartphone digital literacy: general knowledge, skills, motivation, and confidence of using smartphones. These were rated on a scale of 5-point Likert scale, where 1 is the lowest score as “limited” or “not,” 3 as “basic” or “somewhat,” and 5 as “extensive” or “highly.”

##### Skill Assessment

Both pre- and post-course surveys contained twenty-nine statements within five categories of smartphone digital skills: mobile device basics, common apps basics, internet, communication, and security and safety. Participants were asked to rate their competencies on a 5-point Likert scale, where 1 is rated as “no,” 3 is “I think so,” and 5 is “yes.”

##### Evaluation of the Course

The post-course survey contained six questions designed to provide feedback to ACO on the *SilverTech* course:

1. Tell us if the course has met your needs? Yes /No
2. As a result of the course would you like further learning opportunities? Yes /No
3. Did we treat you well? Scale of 5 stars
4. Tell us something you’ll remember about the course? Free-text response (course recall)
5. Any other comments, thoughts on how the course content /tutor could improve the course? Free-text response (course feedback)

The free-text responses were categorized thematically (Braun & Clarke, 2006).

##### Data Analysis

Data were entered manually onto a Microsoft excel spreadsheet by ACO staff and securely stored in Microsoft Teams. The accuracy of data entry was confirmed by a verification check by author JL of a random sample of 10 pre-course surveys and 10 post-course surveys. Data were cleaned, de-identified, and analyzed using STATA software.

A descriptive analysis was used to describe the data on general demographics, digital literacy, and evaluation of the course content. Paired sample t-tests were used to compare the scores of each variable between pre- and post-course surveys for each participant.

## Results

### General Demographics

The demographics of 122 pre-course and 98 post-course survey participants are shown in Table 2. The demographics for each group were comparable, with similar proportions by location, gender, age, ethnicity, and living situation. The remaining data analysis was restricted to the 98 participants with pre- and post-course survey data. The majority of participants attended the South Dunedin and Mosgiel course, reflective of these locations being larger older adult population catchment areas. Three-quarters of participants were female and almost two-thirds lived alone. The majority were aged 76–85 years and identified as New Zealand European.

**Table 2.** Descriptive Characteristics of Participants in SilverTech Course who did Pre- and Post-course Surveys.

	Pre-survey (*n* = 122)	Post-survey (*n* = 98)
Course location (*n*, %)		
Urban		
South Dunedin	30 (25%)	25 (26%)
Dunedin Central	23 (19%)	15 (15%)
Marae	7 (6%)	5 (5%)
Oamaru	9 (7%)	8 (8%)
Rural		
Milton	4 (3%)	3 (3%)
Mosgiel	49 (40%)	42 (43%)
Gender (*n*, %)		
Male	31 (25%)	26 (27%)
Female	91 (75%)	72 (73%)
Age (*n*, %)		
61–65	3 (2%)	2(2%)
66–70	15 (12%)	14 (14.3%)
71–75	24 (20%)	21 (21.4%)
76–80	29 (24%)	24 (24.5%)
81–85	29 (24%)	24 (24.5%)
86+	22 (18%)	13 (13.3%)
Ethnicity (*n*, %)		
NZ European	112 (92%)	92 (94%)
Māori	7 (6%)	4 (4%)
Other	3 (2%)	2 (2%)
Living situation (*n*, %)		
Living alone	78 (64%)	63 (64%)
Living with others	44 (36%)	35 (36%)

### Pre-and Post-Digital Literacy Questions

Table 3 summarizes means and standard deviations of the general assessment and skill assessment digital literacy questions in the pre-course and post-course surveys. The four measures of general assessment of digital literacy increased in the post-course survey, compared with participants’ assessment of their knowledge, skill, motivation, and confidence at baseline (pre-course). Knowledge and skills increased the most after attending the course (*M*_*(post-pre)*_ = 1.35 for knowledge, *M*_*(post-pre)*_ = 1.30 for skills). Motivation for attending was high in the pre-course survey (*M* = 2.10) and showed the least change (*M*_*post-pre*_ = 0.33) compared to the other three variables. With regards to the 29 skill assessment questions, the mean score for each question in the post- course survey (range of mean scores, 2.95–4.45) was higher than the mean score for each equivalent question in the pre-course survey (range of means, 1.54–3.58). For example, the pre-course survey mean score for “I can adjust text font size” was 2.18, and this increased to 3.88 in the post-course survey.

**Table 3.** Means and Standard Deviations of Digital Literacy Questions in Pre-survey and in Post-surveys.

Measures	Pre-survey (*n* = 98)		Post-survey (*n* = 98)	
	Mean	Std. Deviation	Mean	Std. Deviation
Knowledge	2.10	0.96	3.45	0.84
Skill	2.00	0.94	3.30	0.89
Motivation	3.56	1.10	3.89	1.02
Confidence	2.35	0.95	3.47	0.89
Mobile device basics				
I know how to connect to other Wi-Fi connections (e.g., public)	2.00	1.32	3.36	1.30
I can use finger movements (tap, tap/hold, swipe etc)	3.57	1.32	4.26	0.80
I can navigate between screens and pages	2.80	1.32	4.06	0.89
I can recognize navigation icons, for example, search/edit/back button	2.42	1.27	3.97	0.87
I know what a home screen is and its use	3.15	1.32	4.45	0.79
I can adjust text font size	2.18	1.22	3.88	1.12
I can use the on-screen keyboard to type	3.15	1.49	3.95	1.05
I can use the microphone to do voice typing	1.54	0.96	3.23	1.26
Common apps basics				
I can add, edit, or delete a contact on my phone	2.69	1.44	4.14	0.90
I can take photos and find them again	2.93	1.46	4.10	0.96
I know how to use my calendar and add an appointment/event	1.78	1.11	3.66	1.20
I know how to use the clock for alarms and find world times	2.07	1.30	3.94	1.04
I can use the Maps app to find directions to a specific address	1.92	1.08	3.81	1.16
I know how to search for and download a new app	1.72	1.02	3.77	1.08
I can locate, move, or remove apps from my phone	1.70	1.03	3.85	1.05
Internet				
I can log on to the internet on my phone	2.32	1.47	3.71	1.31
I am comfortable going online and searching the internet	2.47	1.44	3.68	1.19
I have some knowledge about shopping online	2.01	1.21	2.95	1.29
I have some knowledge about banking online	2.25	1.48	3.28	1.46
Communication				
I have knowledge about video calling, for example, FaceTime/Messenger/	1.74	1.07	3.12	1.26
What’s App/Skype/Zoom/Google Duo				
I can write an email and attach a photo or file	1.90	1.16	3.34	1.33
I can forward an attach a photo or file by txt	2.23	1.45	3.39	1.29
I can text and send to more than one person at a time	1.95	1.23	3.46	1.31
Security and safety				
I can set up a lock password/pin on my phone	2.06	1.26	3.83	1.08
I have Wi-Fi at home and know where the password is	2.92	1.55	3.90	1.40
I understand Wi-Fi security (locked and unlocked Wi-Fi)	3.58	1.16	3.58	1.26
I have a general understanding of app safety	1.61	0.82	3.68	0.95
I know how to keep safe from scams online	2.01	1.24	3.84	0.93
I know what to do if you suspect you have been caught out by a scammer	2.00	1.22	4.02	0.91

Paired sample t-tests were carried out to determine if these changes in mean scores between pre-course and post-course assessment were significant (Table 4). The paired sample t-tests showed a significant increase in mean scores from pre-course to post-course for the general assessment questions and all 29 skill assessment questions. Participants’ assessment of their skills within the “common apps basics” and “security and safety” domains showed the largest change in mean score. For example “knowing how to download an app” mean scores increased from pre-course (M = 1.72, SD = 1.02) to post-course (M = 3.77, SD = 1.08), t(90) = 15.58, *p* < .001. The mean increase in scores was 2.02 points with a 95% confidence interval ranging from 1.76 to 2.28.

**Table 4.** Paired Sample t-tests to Compare Participants’ Digital Literacy After Attending SilverTech Course.

Measures	n = 98						
	Paired Differences (Post-survey – Pre-survey)				t	df	p
	Mean	Std. deviation	Mean Std. error	95% Confidence Interval of the Difference			
Knowledge	1.35	0.96	0.10	(1.15, 1.54)	13.83	97	<0.001
Skill	1.30	0.90	0.09	(1.12, 1.48)	14.27	97	<0.001
Motivation	0.33	1.16	0.12	(0.09, 0.56)	2.80	97	0.0062
Confidence	1.12	0.91	0.09	(0.94, 1.31)	12.20	97	<0.001
Mobile device basics							
I know how to connect to other Wi-Fi connections (e.g., public)	1.36	1.35	1.15	(1.07, 1.65)	9.38	85	<0.001
I can use finger movements (tap, tap/hold, swipe etc)	0.67	1.16	0.12	(0.43, 0.91)	5.61	93	<0.001
I can navigate between screens and pages	1.23	1.19	1.13	(0.97, 1.48)	9.66	87	<0.001
I can recognize navigation icons, for example., search/edit/back button							
I know what a home screen is and its use	1.32	1.22	1.13	(1.07, 1.58)	10.31	89	<0.001
I can adjust text font size	1.68	1.39	1.15	(1.38, 1.98)	11.15	84	<0.001
I can use the on-screen keyboard to type	0.85	1.35	1.15	(0.56, 1.14)	5.79	84	<0.001
I can use the microphone to do voice typing	1.65	1.29	1.14	(1.37, 1.93)	11.64	82	<0.001
Common apps basics							
I can add, edit, or delete a contact on my phone	1.44	1.26	1.13	(1.18, 1.70)	10.92	90	<0.001
I can take photos and find them again	1.13	1.54	1.16	(0.81, 1.45)	7.07	92	<0.001
I know how to use my calendar and add an appointment/event	1.85	1.20	1.13	(1.60, 2.11)	14.55	88	<0.001
I know how to use the clock for alarms and find world times	1.88	1.23	1.13	(1.63, 2.14)	14.73	92	<0.001
I can use the Maps app to find directions to a specific address	1.89	1.13	1.12	(1.66, 2.12)	16.19	92	<0.001
I know how to search for and download a new app	2.02	1.24	1.13	(1.76, 2.28)	15.58	90	<0.001
I can locate, move, or remove apps from my phone	2.07	1.20	1.13	(1.81, 2.32)	16.10	86	<0.001
Internet							
I can log on to the internet on my phone	1.40	1.44	1.15	(1.10, 1.69)	9.27	90	<0.001
I am comfortable going online and searching the internet	1.15	1.31	1.14	(0.88, 1.42)	8.44	91	<0.001
I have some knowledge about shopping online	0.91	1.13	1.12	(0.68, 1.15)	7.69	90	<0.001
I have some knowledge about banking online	0.96	1.08	1.11	(0.73, 1.18)	8.41	90	<0.001
Communication							
I have knowledge about video calling, for example, FaceTime/Messenger/What’s App/Skype/Zoom/Google Duo	1.36	1.19	1.12	(1.12, 1.61)	10.94	90	<0.001
I can write an email and attach a photo or file	1.47	1.25	1.13	(1.21, 1.72)	11.38	93	<0.001
I can forward an attach a photo or file by txt	1.16	1.23	1.13	(0.91, 1.41)	9.12	92	<0.001
I can text and send to more than one person at a time	1.47	1.47	1.16	(1.16, 1.78)	9.34	86	<0.001
Security and safety							
I can set up a lock password/pin on my phone	1.77	1.45	1.16	(1.46, 2.09)	11.21	83	<0.001
I have Wi-Fi at home and know where the password is	0.92	1.40	1.15	(0.63, 1.22)	6.21	88	<0.001
I understand Wi-Fi security (locked and unlocked Wi-Fi)	1.63	1.49	1.31	(1.31, 1.95)	10.15	85	<0.001
I have a general understanding of app safety	2.07	1.04	1.11	(1.85, 2.28)	18.92	90	<0.001
I know how to keep safe from scams online	1.79	1.19	1.12	(1.55, 2.04)	14.44	91	<0.001
I know what to do if you suspect you have been caught out by a scammer	2.00	1.15	1.12	(1.76, 2.24)	16.48	89	<0.001

### Evaluation of Course

Responses to the yes/no questions (Table 5) indicated that the majority (95%) of participants considered that the *SilverTech* course they attended had met their needs. Seventy percent of participants indicated that they were interested in further learning opportunities.

**Table 5.** Participants’ Assessment of the SilverTech Course: Needs Met, Further Learning, and Treatment.

Measures	Post-survey (n = 98)
Course met needs	
Yes (n, %)	95 (96.94%)
No (n, %)	3 (3.06%)
Like further learning	
Yes (n, %)	69 (70.41%)
No (n, %)	29 (29.59%)
Treated well (mean ± SD)	4.94 ± 0.32

Text feedback about the course included comment on the “excellence” of the tutors and handouts, appreciation of clear instructions, and appreciation of the small size group learning.

“Great course, tutor is very relaxed and confident.”

“I liked there being small numbers so tutor could spend time on a one-to-one basis.”

“Well worthwhile. Best course I have ever been on. Great handouts to look back on.”

Suggestions for improvement to the course included providing refresher sessions, more one on one time with tutors, and expanding course content to cover tablets, and using Excel spreadsheets.

## Discussion

The results indicate that the *SilverTech* course improved older adults’ smartphone digital literacy. Participants reported improved levels of knowledge, skills, motivation to try new applications, and confidence in smartphone use. The significant increase in mean scores for the 29 skills assessed suggests that the four-week (8 hour) small group course was effective. Alongside reported improvement in a range of smartphone skills, participant feedback showed that they valued the course design—small class size, take-home handouts, and opportunities for one-to-one tutorage—and the majority indicated a desire for further training and learning opportunities.

Our results align with those of previous research (Delello & McWhorter, 2017; Lu, Wen et al., 2017) in demonstrating that bespoke short educational programs contribute to improving digital literacy among older adults. Participants’ recall and feedback of the *SilverTech* courses were very positive, and this affirms previous research indicating that educational courses with small classes that provide a combination of hands-on tutorials and one-on-one teaching with printed take-home hand-outs suit the pedagogical needs of older adults (Flauzino et al., 2020). While Martínez-Alcalá et al. (2018) reported that blended learning workshops (using a Learning Management System [LMS] digital platform) were slightly more effective at increasing digital literacy using computers than face to face workshops, this may not be applicable with improving digital literacy through smartphones and tablets. The advantage of printed hand-outs is that they can be taken away for future reference and are not dependent on a threshold of digital literacy to access the LMS platform away from the course.

The findings indicate that courses with the aim of improving older adults’ digital literacy and fluency must be realistic and pragmatic. In the present study, most participants reported high levels of satisfaction with the course which suggests that the content was considered relevant. Shreurs et al. (2017) noted that while their participants gained competencies on navigating digital devices, understanding system operation knowledge (which is often necessary for problem shooting and applying fixes) was not so relevant. They suggested this gap can be mitigated by having appropriate supports in place (Schreurs et al., 2017). Developers of digital literacy courses for older adults need to monitor the relevance of course content and check that participants have a “digital tech buddy” able to assist as required—this might be a younger family member. For older adults in retirement villages or residential aged care facilities, this could be a staff member. Support systems in training programs have been shown to mitigate the influence of socio-demographic factors of older adults such as income, employment status, and level of education (Tirado-Morueta, Aguaded-Gómez et al., 2018). In the present study, the majority of participants lived alone and did not have access to support from other household members. Online tutorial videos are currently being developed for the *SilverTech* program and will be available to all graduates. The next phase of the *SilverTech* course development could include drop-in support sessions for older adults who have previously completed the course, thus meeting the need for ongoing training identified by participants.

Manor and Herscovici (2021) argued that digital ageism is apparent in the exclusion of older adults by developers of devices and digital apps because of stereotypical presumptions that mean the unique needs of older adults are not recognized. Smartphones and tablets offer older adults a portal to the digital world that is more accessible than computers. But device developers have yet to recognize the market potential of this growing demographic (Manor & Herscovici, 2021; McDonough & Kingsley, 2015; Schreurs et al., 2017; Tsai et al., 2017). Marcelino et al. (2015) suggested that developers should use design interfaces that older adults are likely to already be familiar with and this might involve removing double clicks, having large interface buttons, either no scrolling or slower scrolling, and to minimize how many clicks are necessary to complete tasks. Pull down menus should be short, font type and size need to be large, and exit options need to be obvious (Marcelino et al., 2015).

The positive impacts of digital devices on older adults’ well-being and health are well documented (Marcelino et al., 2015; Zolbin et al., 2022). Apps already exist for appointment, medication and routine reminders, remote supervision and monitoring, alarming and emergency contacts, and sharing or storing data, and their potential application to support activities of daily living continues to grow (McDonough & Kingsley, 2015; Susło et al., 2018). Alongside improving accessibility for the general older adult population, smartphone apps have substantial benefits for reducing inequities experienced by older adults with disabilities. Lips et al. (2020) reported that older NZ adults with disabilities or mobility/sensory impairments found digital apps to be of great assistance and utility. It has been suggested that improved digital literacy could have system level benefits also and potentially reduce demands on healthcare systems (Tsai et al., 2017; Zolbin et al., 2022). For example, the GPS trackers on smartphones provide caregivers of people with dementia the ability to track them. Digital devices can empower older adults by supporting independence and social participation (Barrie et al., 2021), access to information (Tirado-Morueta et al., 2018), and entertainment. Further research is needed to explore the utility and user experience of these apps with older adults.

A critical barrier to the uptake of digital technology is the fear of online fraud. Although Kemp and Erades Pérez (2023) found that older adults are no more likely to suffer online fraud than other age groups, older adults feel particularly vulnerable to digital fraud (Lips et al., 2020). Online safety campaigns and information struggle to keep up with the increasing sophistication of scammers (Hageman, 2022). In the New Zealand context, the NGO Netsafe provides advice on internet and cyber safety, while NGOs such as SeniorNet and ACO are also actively promoting digital safety through their training courses. The interest declared by 70% of participants in the present study in further learning opportunities suggests that refresher courses may be well attended, and these would provide an opportunity for older adults to keep up with digital security while maintaining their digital literacy confidence.

New Zealand has invested in a national ultrafast fiber broadband network and is among world leaders in internet service infrastructure cover. But the affordability of access to the internet (Digital Equity Coalition Aotearoa, 2023), and upgrading devices and software remains an issue (Lips et al., 2020]). As government and other services are digitized, governments have a responsibility to ensure that citizens are not marginalized by digital exclusion. The NZ government recently announced a three-year funding package for digital skills courses for older adults (Andersen, 2023). With an aging population and fast-paced technological advances (such as AI), funding needs to be available beyond the three years for the digital literacy gains to be maintained/retained.

### Limitations

There are several limitations to this study. The research population did not reflect the diversity of the New Zealand population in terms of ethnicity —the majority of participants being female and NZ European. A research sample with greater demographic diversity may have impacted the findings of the present study. It is possible that the needs of Māori, Pacific, and Asian older adults for smartphone training are being met by other organizations. In the event of the ACO *SilverTech* course being extended over NZ, considerations need to be given to identifying/ implementing strategies to ensure the course reaches and meets the needs of the ethnically diverse older adult population in New Zealand. The lack of follow-up beyond the post-course survey at the completion of the course means we have no knowledge of skill retention and confidence over a longer period of time. Similarly, our findings are based upon self-reported assessment of ability and skills. Future evaluations could be strengthened by an objective assessment of skills.

## Conclusion

This study set out to evaluate the effectiveness of the ACO *SilverTech* course with a view to extending it across the southern region of NZ and ultimately expanding nationally across NZ. The findings affirm that the course is meeting participants’ needs, was effective at improving digital literacy, and stimulating desire for further training. Further research needs to be conducted to explore the balance between the domains of knowledge, confidence, and technical skills as older adults continue to age (often with decreasing functional capability), against the rapid development of digital technology. The rapid pace of technological innovation may result in similar gray divides in future generations because it cannot be assumed that the current digital competencies of younger cohorts will protect them from lagging behind technological developments and innovations as they age. Attention to understanding post-digital literacy course retention of skills over longer periods of time would be beneficial to identify content for refresher courses.
